# Prevalence of gout and asymptomatic hyperuricemia in the pediatric population: a cross-sectional study of a Japanese health insurance database

**DOI:** 10.1186/s12887-020-02379-0

**Published:** 2020-10-15

**Authors:** Shuichi Ito, Tomoko Torii, Akihiro Nakajima, Takeshi Iijima, Hiroshi Murano, Hideki Horiuchi, Hisashi Yamanaka, Masataka Honda

**Affiliations:** 1 Department of Pediatrics, Graduate School of Medicine, Yokohama City University, Fukuura 3-9, Kanazawa-ku, Yokohama-shi, Kanagawa 236-0004 Japan; 2 Medical Science Department, Teijin Pharma Limited, Kasumigaseki Common Gate West Tower, Kasumigaseki 3-2-1, Chiyoda-ku, Tokyo, 100-8585 Japan; 3 Pharmaceutical Development Administration Department, Teijin Pharma Limited, Kasumigaseki Common Gate West Tower, Kasumigaseki 3-2-1, Chiyoda-ku, Tokyo, 100-8585 Japan; 4 Pharmaceutical Development Coordination Department, Teijin Pharma Limited, Kasumigaseki Common Gate West Tower, Kasumigaseki 3-2-1, Chiyoda-ku, Tokyo, 100-8585 Japan; 5 Rheumatology, Sanno Medical Center, Akasaka 8-5-35, Minato-ku, Tokyo, 107-0052 Japan; 6 Clinical Research Support Center, Tokyo Metropolitan Children’s Medical Center, Musashidai 2-8-29, Fuchu-shi, Tokyo, 183-8561 Japan

**Keywords:** Database study, Real-world prevalence, Gout, Asymptomatic hyperuricemia, Pediatric, Gouty arthritis

## Abstract

**Background:**

Although gout is rare in children, chronic sustained hyperuricemia can lead to monosodium urate deposits progressing to gout, just as in adults. This study assessed prevalence and characteristics of gout and asymptomatic hyperuricemia, and incidence of gouty arthritis in the pediatric population, using data from Japanese health insurance claims. The diagnosis and treatment of pediatric gout and hyperuricemia were analyzed, and specific characteristics of those patients were assessed. Since Japanese guidelines recommend treatment with uric acid lowering drugs for asymptomatic hyperuricemia as well as for gout, these data were also used to investigate the real-world use of uric acid lowering drugs in a pediatric population.

**Methods:**

This cross-sectional study was based on a 2016–2017 Japanese health insurance claims database, one of the largest epidemiology claims databases available in Japan, which included 356,790 males and 339,487 females 0–18 years of age. Outcomes were measured for prevalence, patient characteristics, treatment with uric acid lowering drugs for gout and asymptomatic hyperuricemia, and prevalence and incidence of gouty arthritis. Because uric acid can be elevated by some forms of chemotherapy, data from patients under treatment for malignancies were excluded from consideration.

**Results:**

Total prevalence of gout and asymptomatic hyperuricemia in 0–18 year-olds was 0.040% (276/696,277 patients), with gout prevalence at 0.007% (48/696,277) and asymptomatic hyperuricemia at 0.033% (228/696,277). Prevalence of gout and asymptomatic hyperuricemia was highest in adolescent males, at 0.135% (176/130,823). The most common comorbidities for gout and asymptomatic hyperuricemia were metabolic syndrome at 42.8% (118/276) and kidney disease at 34.8% (96/276). Of the patients diagnosed with gout or asymptomatic hyperuricemia, 35.1% (97/276) were treated with uric acid lowering drugs. Gouty arthritis developed in 43.8% (21/48) of gout patients during the study, at an incidence of 0.65 flares/person-year.

**Conclusions:**

Even the pediatric population could be affected by asymptomatic hyperuricemia, gout, and gouty arthritis, and uric acid lowering drugs are being used in this population even though those drugs have not been approved for pediatric indications. Such off-label use may indicate a potential need for therapeutic agents in this population.

**Trial registration:**

UMIN000036029.

## Background

Gout is an inflammatory condition that is attributable to monosodium urate deposits resulting from hyperuricemia, which is defined as serum uric acid level > 7.0 mg/dL [1, 2].

In pediatric patients, chronic hyperuricemia is often associated with underlying comorbidities such as cardiovascular disease or kidney disease, inborn errors of purine metabolism, genetic disorders, or kidney transplantation [2–13]. Gout is relatively rare in the pediatric population, but diagnostic studies with dual energy computed tomography technology have shown that chronic sustained hyperuricemia in children can lead to monosodium urate deposits that may progress to gout, just as in adults [14–16]. Recent trends have shown that child obesity is increasing, which suggests a possible correlation to the prevalence of gout and hyperuricemia in pediatric patients and which parallels the increasing incidence of gouty arthritis at younger ages [17–19]. For example, Kato et al. have reported the increasing incidence of gouty arthritis in teenagers [20]. However, to our knowledge no studies have been performed on the prevalence, patient characteristics and concomitant diseases, or treatment status for hyperuricemia and gout in pediatric patients.

Data are also needed on the most effective approach for managing asymptomatic hyperuricemia in pediatric populations. The Japanese guideline for the management of hyperuricemia and gout recommends treatment with uric acid lowering drugs, not only for gout but also for asymptomatic hyperuricemia, but in Europe and the US these drugs are recommended for gout only [21–23]. The Japanese guideline recommends that asymptomatic hyperuricemia should first be treated with lifestyle guidance, but that regardless of lifestyle changes, urate lowering therapy should subsequently be considered in patients without comorbidities whose uric acid levels are 9.0 mg/dL or above and in patients with comorbidities such as urinary calculus, kidney disease, hypertension, ischemic heart disease, diabetes, or metabolic syndrome whose uric acid levels are 8.0 mg/dL or above. This recommendation is supported by studies which show that higher uric acid levels correlate with a higher risk of developing gout later in life [24] and that uric acid lowering drugs decreased the incidence of gouty arthritis in patients with asymptomatic hyperuricemia and chronic kidney disease [25]. These findings emphasize the importance of collecting accurate and reliable information on the diagnosis and treatment of hyperuricemia and gout among both adults and children. Such information will enable researchers to understand actual clinical practices within the pediatric population and to identify patients who are at risk for these conditions.

In Japan hyperuricemia is diagnosed as a disease, so Japanese health insurance databases contain information about asymptomatic hyperuricemia. We were able to access one of these databases, which allowed us to assess the diagnosis rates, demographics, and treatment for gout and asymptomatic hyperuricemia in Japanese pediatric patients.

## Methods

### Study design

This retrospective cross-sectional study used medical insurance claims data from April 2016 to March 2017 and was designed to investigate the prevalence of gout and asymptomatic hyperuricemia in the Japanese pediatric population as well as to assess patient characteristics and treatment. The study was registered through the UMIN Clinical Trials Registry (UMIN000036029, February 27, 2019). Data were obtained from the JMDC Claims Database, which is one of the largest epidemiologic insurance claims databases available in Japan and which contained information on approximately 2% of the Japanese population at the time of this study [26]. The health insurance system in Japan is a “universal health insurance coverage system,” which means that all Japanese citizens are covered by some form of comprehensive medical insurance. The fee for each treatment is the same across the country. The co-payment ratio is normally 30% of the total medical cost. Every citizen can visit any medical facility at any time. All people in Japan are insured either through their employer or through a government agency. The JMDC claims database is sourced from multiple health insurance organizations, most of which serve company employers, so this database represents company employees and their dependents.

The JMDC Claims Database has acquired anonymized information from multiple health insurance organizations, along with permission to share some of that information with third parties for research. Data include diagnostic codes, physical examination data, and drug prescription records. Patients are anonymized and assigned identification numbers, which can be traced through multiple hospitals and clinical facilities. The age distribution of pediatric subscribers was identical to that of the overall pediatric population of Japan in 2016–2017.

### Participants

Inclusion criteria for patients in this study were

1. Uninterrupted registration in the JMDC Claims Database from April 2016 to March 2017.
2. Diagnosis of gout or asymptomatic hyperuricemia.
3. Age 0–18 years as of April 2016.

Patients with malignant tumors were excluded, since chemotherapy-induced hyperuricemia would be a confounder.

The definition of “target population” in this study is described in Additional file 1: Table S1.

### Study measures

The following factors were analyzed.

1. Prevalence of gout and asymptomatic hyperuricemia.
2. Patient characteristics such as sex, age, comorbidities, prescription drugs including uric acid lowering drugs, number of serum uric acid measurements per year, and size of the facility where patients were diagnosed.
3. Prevalence and incidence of gouty arthritis.

The terms “comorbidities,” “prescription drugs” including “uric acid lowering drugs,” and “gouty arthritis” in this study are defined in Additional file 1: Table S1.

### Statistical methods

Because this was a cross-sectional study to investigate actual clinical situations, sample size was not calculated. All insurance subscribers who met the eligibility criteria were included in the analysis. Patient characteristics and prevalence were explored with descriptive statistical methods. Prevalence was calculated overall and by age group and sex using the following formula:

$$ \mathrm{Prevalence}=\frac{\mathrm{Number}\kern0.17em \mathrm{of}\kern0.17em \mathrm{patients}\kern0.17em \mathrm{diagnosed}\kern0.17em \mathrm{with}\kern0.17em \mathrm{gout}\kern0.17em \mathrm{or}\kern0.17em \mathrm{asymptomatic}\kern0.17em \mathrm{hyperuricemia}}{\mathrm{Number}\kern0.17em \mathrm{of}\kern0.17em \mathrm{subscribers}\kern0.17em \mathrm{in}\kern0.17em \mathrm{pediatric}\kern0.17em \mathrm{population}} $$

The incidence of gouty arthritis in gout patients, in gout and asymptomatic hyperuricemia patients, and in the total pediatric subscriber population was calculated overall and for each age group, and was assessed as the incidence rate. The numerator was the total number of gouty arthritis events during a single year from April 2016 to March 2017 in patients who had been diagnosed with gout.

$$ \mathrm{Incidence}\kern0.17em \mathrm{rate}=\frac{\mathrm{Total}\kern0.17em \mathrm{number}\kern0.17em \mathrm{of}\kern0.17em \mathrm{occurrences}\kern0.34em \mathrm{of}\kern0.17em \mathrm{gouty}\kern0.17em \mathrm{arthritis}}{\mathrm{Total}\kern0.17em \mathrm{duration}\kern0.17em \mathrm{of}\kern0.17em \mathrm{follow}\hbox{-} \mathrm{up}\;\left(\mathrm{person}\hbox{-} \mathrm{year}\right)} $$

For each continuous variable, mean and standard deviation (SD) were calculated for data in a normal distribution, and for median and interquartile range (IQR) if it was not a normal distribution. Categorical variables were summarized by number and percentage of patients.

All analyses were performed using SAS version 9.4.

## Results

### Study population

**Fig. 1:**
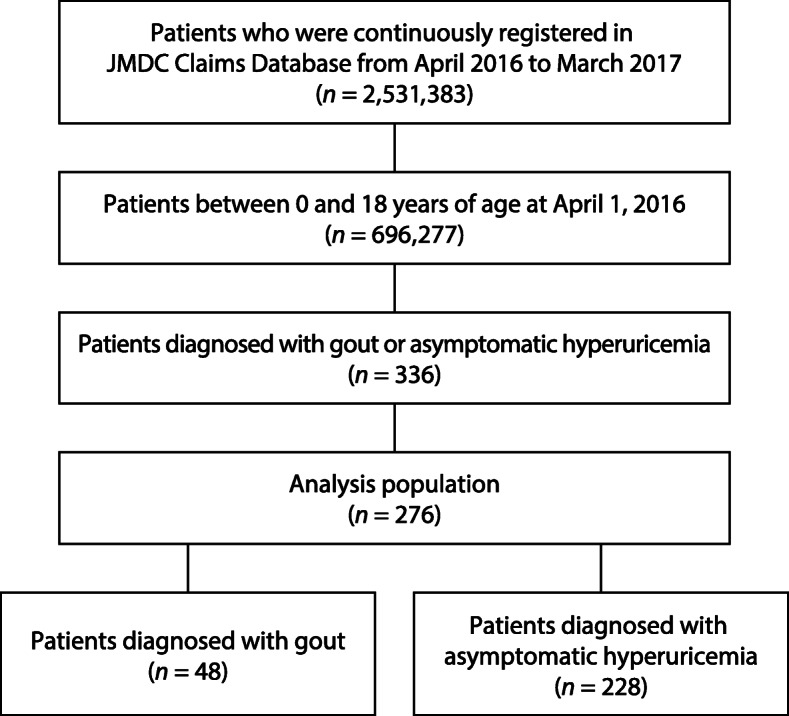
Disposition of patients in the analysis set

A total of 696,277 children (356,790 male and 339,487 female) were 0 to 18 years of age as of April 1, 2016 and were registered in the JMDC Claim Database without interruption throughout the study period. Within that group, 276 (209 male and 67 female) were diagnosed with gout or asymptomatic hyperuricemia: 48 with gout (33 male and 15 female) and 228 with asymptomatic hyperuricemia (176 male and 52 female) (Fig. 1).

### Prevalence of gout and asymptomatic hyperuricemia by age group

The overall prevalence of gout and asymptomatic hyperuricemia from 0 to 18 years old was 0.040% (276/696,277). Prevalence was 0.007% (48/696,277) for gout alone and 0.033% (228/696,277) for asymptomatic hyperuricemia, which was higher than for gout (Table 1).

**Table 1:** Number of patients diagnosed with gout or asymptomatic hyperuricemia

	Total population	Gout and asymptomatic hyperuricemia	Gout	Asymptomatic hyperuricemia
0–18 years				
All, *n* (%)	696,277	276 (0.040)	48 (0.007)	228 (0.033)
Male, *n* (%)	356,790	209 (0.059)	33 (0.009)	176 (0.049)
Female, *n* (%)	339,487	67 (0.020)	15 (0.004)	52 (0.015)
0–5 years				
All, *n* (%)	220,749	22 (0.010)	3 (0.001)	19 (0.009)
Male, *n* (%)	113,406	10 (0.009)	1 (< 0.001)	9 (0.008)
Female, *n* (%)	107,343	12 (0.011)	2 (0.002)	10 (0.009)
6–11 years				
All, *n* (%)	219,509	43 (0.020)	7 (0.003)	36 (0.016)
Male, *n* (%)	112,561	23 (0.020)	2 (0.002)	21 (0.019)
Female, *n* (%)	106,948	20 (0.019)	5 (0.005)	15 (0.014)
12–18 years				
All, *n* (%)	256,019	211 (0.082)	38 (0.015)	173 (0.068)
Male, *n* (%)	130,823	176 (0.135)	30 (0.023)	146 (0.112)
Female, *n* (%)	125,196	35 (0.028)	8 (0.006)	27 (0.022)

For gout and asymptomatic hyperuricemia, prevalence by age group was 0.010% (22/220,749) at 0–5 years of age, 0.020% (43/219,509) at 6–11 years of age, and 0.082% (211/256,019) at 12–18 years of age, indicating higher prevalence with age. This change in prevalence at adolescence was more pronounced in males than in females (0.135% [176/130,823] in males and 0.028% [35/125,196] in females for gout and asymptomatic hyperuricemia in subjects 12–18 years of age) (Table 1).

### Patient characteristics

Median age (IQR) for pediatric gout and asymptomatic hyperuricemia patients was 15.0 (12.0, 17.0) years; 75.7% (209/276) were male and 24.3% (67/276) were female. Notably, the distribution of gout and hyperuricemia was similar in both sexes at age 0–11. However, at age 12–18, distribution shifted to 83.4% (176/211) males and 16.6% (35/211) females (Table 2).

**Table 2:** Patient characteristics for diagnosed gout and asymptomatic hyperuricemia

	Total (*n* = 276)	Age		
		0–5 years (*n* = 22)	6–11 years (*n* = 43)	12–18 years (*n* = 211)
Age, years				
Median [Quartile]	15.0 [12.0, 17.0]	3.5 [2.0, 4.0]	9.0 [8.0, 11.0]	16.0 [14.0, 17.0]
Sex, *n* (%)				
Male	209 (75.7)	10 (45.5)	23 (53.5)	176 (83.4)
Female	67 (24.3)	12 (54.5)	20 (46.5)	35 (16.6)
Comorbidity, *n* (%)				
Kidney disease	96 (34.8)	6 (27.3)	14 (32.6)	76 (36.0)
Cardiovascular disease	63 (22.8)	9 (40.9)	15 (34.9)	39 (18.5)
Metabolic syndrome	118 (42.8)	5 (22.7)	20 (46.5)	93 (44.1)
Down syndrome	15 (5.4)	2 (9.1)	3 (7.0)	10 (4.7)
Treatment and drug use, *n* (%)				
Cardiovascular disease drug	9 (3.3)	0 (0.0)	5 (11.6)	4 (1.9)
Diuretic drug	13 (4.7)	2 (9.1)	4 (9.3)	7 (3.3)
β blocker	7 (2.5)	1 (4.5)	2 (4.7)	4 (1.9)
Ca antagonist	8 (2.9)	0 (0.0)	1 (2.3)	7 (3.3)
ACE inhibitor and/or ARB	32 (11.6)	1 (4.5)	6 (14.0)	25 (11.8)
Antihyperlipidemic drug	14 (5.1)	0 (0.0)	0 (0.0)	14 (6.6)
Antidiabetic drug	5 (1.8)	0 (0.0)	0 (0.0)	5 (2.4)
Immunosuppressant	18 (6.5)	1 (4.5)	7 (16.3)	10 (4.7)
Vitamin D	13 (4.7)	0 (0.0)	3 (7.0)	10 (4.7)
Dialysis	2 (0.7)	0 (0.0)	0 (0.0)	2 (0.9)
Treatment with uric acid lowering drug, *n* (%)	97 (35.1)	1 (4.5)	10 (23.3)	86 (40.8)
Number of measurement of sUA				
Median [Quartile]	2.0 [1.0, 4.0]	1.0 [0.0, 3.0]	3.0 [1.0, 6.0]	2.0 [1.0, 5.0]
Size of the facility, *n* (%)				
0–19 beds	113 (40.9)	3 (13.6)	11 (25.6)	99 (46.9)
20–99 beds	6 (2.2)	1 (4.5)	0 (0.0)	5 (2.4)
100–299 beds	31 (11.2)	2 (9.1)	4 (9.3)	25 (11.8)
≥ 300 beds	131 (47.5)	16 (72.7)	28 (65.1)	87 (41.2)

*ACE* Angiotensin-converting enzyme, *ARB* Angiotensin II receptor blocker, *sUA* Serum uric acid

The most common comorbidities for gout and asymptomatic hyperuricemia were metabolic syndrome (42.8% [118/276]), followed by kidney disease (34.8% [96/276]). Although all age groups included a similar percentage of patients with kidney disease, advancing age was associated with higher rates of comorbid metabolic syndrome. Fifteen patients had comorbid Down syndrome (Table 2), and a wide variety of other comorbidities including gastroenteritis and respiratory infection were also noted (Additional file 1: Table S2). Some patients had multiple comorbidities (data not shown).

In this study, angiotensin-converting enzyme (ACE) inhibitors and/or angiotensin II receptor blockers (ARBs) were used by 11.6% (32/276) of patients, immunosuppressants by 6.5% (18/276), and diuretics by 4.7% (13/276). Within the overall patient population, 35.1% (97/276) were prescribed uric acid lowering drugs. By age group, those numbers were 4.5% (1/22) for 0–5 years of age, 23.3% (10/43) for 6–11 years of age, and 40.8% (86/211) for 12–18 years of age (Table 2).

The median number (IQR) of serum uric acid measurements per year was 2.0 (1.0, 4.0). This median number varied widely among the age groups; the highest was 3.0 (1.0, 6.0) measurements per year in patients 6–11 years of age. We also surveyed the types of medical facilities at which the patients were diagnosed: 40.9% (113/276) were seen at clinics with 0–19 beds, and 47.5% (131/276) at large hospitals with 300 beds or more. By age group, data showed that a higher proportion of younger children were diagnosed at large hospitals, and that a higher proportion of older pediatric patients were diagnosed at small hospitals or clinics (Table 2).

### Prevalence and incidence of gouty arthritis

Among the 48 patients who were identified as having gout, 21 had gouty arthritis during the study period (Table 3). The prevalence of gouty arthritis was 43.8%, and the incidence of gouty arthritis was 0.65 flares/person-year. Three patients 0–5 years of age were diagnosed with gout but did not have gouty arthritis. Gouty arthritis occurred in 3 of 7 (42.9%) patients 6–11 years of age (1.43 flares/person-year) and 18 of 38 (47.4%) patients 12–18 years of age (0.55 flares/person-year). Among the total number of patients diagnosed with gout or asymptomatic hyperuricemia during the study period, the prevalence of gouty arthritis was 7.0% (3/43) in patients 6–11 years of age and 8.5% (18/211) in patients 12–18 years of age.

**Table 3:** Number of patients and prevalence of gouty arthritis (patients with events during the period April 2016 to March 2017)

	Total	Age		
		0–5 years	6–11 years	12–18 years
Number of patients with gouty arthritis				
*n*	21	0	3	18
Prevalence of gouty arthritis, % (*n/N*)				
In patients with gout	43.8 (21/48)	0 (0/3)	42.9 (3/7)	47.4 (18/38)
95% CI	29.5–58.8	0.0–70.8	9.9–81.6	31.0–64.2
In patients with gout and asymptomatic hyperuricemia	7.6 (21/276)	0 (0/22)	7.0 (3/43)	8.5 (18/211)
Within the database population	0.003 (21/696,277)	0 (0/220,749)	0.001 (3/219,509)	0.007 (18/256,019)

*CI* Confidence interval

Of the 21 patients who developed gouty arthritis during the study period, uric acid lowering drugs were prescribed to 6 patients, colchicine to 4 patients, nonsteroidal antiinflammatory drugs to 20 patients, and oral steroids to 2 patients.

## Discussion

To our knowledge, this is the first cross-sectional study of gout and asymptomatic hyperuricemia in children and adolescents. The findings clearly demonstrate the prevalence, demographics, and clinical treatment practices for gout and hyperuricemia in this population.

Children generally have lower serum uric acid levels than adults, so the prevalence of gout and asymptomatic hyperuricemia is assumed to also be lower in the pediatric population than in adults [3]. Previously, Koto et al. performed a similar cross-sectional study of the prevalence of gout and asymptomatic hyperuricemia in Japanese adults using the JMDC database; those results showed prevalence in adults of 1.1% for gout and 2.6% for asymptomatic hyperuricemia [27]. In the present study, the prevalence of gout in the pediatric population, aged 0–18 years, was found to be approximately 1/100 of that in the adult population.

In men, testosterone is thought to increase uric acid level, while in women, estrogen is thought to increase uric acid excretion. Women thus typically have lower uric acid levels than men [2]. These trends are confirmed by our findings for prevalence by age group and by sex in this study. We estimated that sex differences would continue from adolescence into adulthood.

A variety of underlying congenital, genetic, and chronic diseases have been reported in pediatric patients who have chronic persistent hyperuricemia, including cyanotic congenital heart disease and inborn errors of metabolism [2–4]. Such hyperuricemia can also be complicated by congenital abnormalities of kidney form and function, by nephrotic syndrome, and by kidney transplantation [3, 11–13]. Abnormal purine metabolism, in conditions such as Lesch-Nyhan syndrome or inborn errors of metabolism, is associated with hyperuricemia [3], and early onset of hyperuricemia and of gout have also been reported in children with Down syndrome [9, 10]. The results of this study confirmed previous reports of patients with underlying chronic diseases including cyanotic congenital heart disease, kidney disease, and Down syndrome [2, 3]. In our study, no patients with Lesch-Nyhan syndrome were identified in the database, perhaps because of the rarity of this disease (prevalence 1/380,000) [5]. The prevalence of comorbidities was documented in detail in Table S2. Notably, most of those conditions were considered to be typical of diseases seen in children. In Japan, when pediatric patients visit medical institutions and undergo blood biochemical tests, a range of items are measured, including serum uric acid levels in many cases. Such measurements are covered by national health insurance, and hyperuricemia is diagnosed when an increase in uric acid levels is observed. Children are known to have a higher frequency of secondary hyperuricemia than adults due to comorbidities or related conditions. For example, gastroenteritis is accompanied by dehydration, resulting in transient hyperuricemia [3], and it is possible that the effects of transient hyperuricemia may be observed in blood biochemical tests for various comorbidities in pediatric patients in Japan.

Results, arranged by age group and medical facilities, showed that younger children tended to be diagnosed at larger hospitals. Those young patients were presumed to have hyperuricemia secondary to underlying diseases such as congenital cardiovascular or kidney disease. However, older pediatric patients tended to be diagnosed at smaller facilities. A relationship between increasing age and the development of metabolic syndrome and associated diseases was also observed. That relationship may explain the tendency toward diagnosis at smaller facilities for older pediatric patients. Other research supports these findings, showing a positive correlation between obesity and high uric acid level and indicating that hyperuricemia in childhood and adolescence is associated with higher risk for lifestyle-related diseases [28, 29]. Such findings have led to growing interest in the relationship between metabolic syndrome and hyperuricemia during puberty.

This study showed an age-related increase in the number of cases of kidney disease, cardiovascular disease, and/or Down syndrome as comorbidities of hyperuricemia. Those findings may be explained by gradual increases in serum uric acid level as a result of comorbidities from a young age, rather than by the onset of new comorbidities. Serum uric acid levels also increase due to elevated testosterone at puberty, and the combination of long-standing comorbidities and the onset of adolescence may have triggered hyperuricemia.

The relatively high percentage of patients with hyperuricemia in this study who were treated with ACE inhibitors or ARBs may reflect an abnormally high prevalence of cardiovascular and renal disease in this population. It is unclear whether ACE inhibitors or ARBs themselves have a direct effect on serum uric acid levels.

Overall, 35.1% of patients were treated with uric acid lowering drugs. Findings from this study suggest that uric acid lowering drugs are being prescribed for a relatively high proportion of asymptomatic hyperuricemia patients as well as for gout patients. These findings may reflect the recommendation of the Japanese Society of Gout and Nucleic Acid Metabolism for the use of uric acid lowering drugs to treat asymptomatic hyperuricemia patients, despite the fact that pediatric formulations are not yet available, while in Western countries this treatment is limited to patients who suffer from gouty arthritis [21–23].

Our study showed that gouty arthritis occurred in 43.8% of pediatric patients with gout and in at least 7.0% of the combined population of pediatric patients with gout or asymptomatic hyperuricemia. These findings should increase awareness of the risk of gouty arthritis among pediatric patients with gout or asymptomatic hyperuricemia. The introduction of uric acid lowering drugs should be considered in such pediatric patients. But these drugs have not yet been approved for pediatric indications. There is an unmet need for the development of uric acid lowering drugs that are safe and effective in pediatric patients.

This study has several limitations. First, it was a cross-sectional study that was based on information from insurance claims (medical fee claim forms) for treatment, so the validity of definitions for various diseases, including gout, was not assured. Second, slightly fewer than 300 pediatric patients were identified as having gout or asymptomatic hyperuricemia, and only limited discussion is possible for aggregated results from categories with such a small number of subjects. Third, these data were from company employees and their family members, which excluded people who were not working in corporations and their families, reducing the generalizability of our findings. Fourth, some patients had claims reimbursed by a bundled payment plan, and only limited information may have been available on prescriptions and laboratory tests for those patients. Fifth, this was a cross-sectional study with evaluation at a single time point, so it was not possible to estimate the causal relationship between exposure and outcome.

This article reports the proportion of pediatric patients with a diagnosis of gout or asymptomatic hyperuricemia who received drug therapy with uric acid lowering drugs. We investigated actual treatment protocols with uric acid lowering drugs for those patients in a separate study, to be reported in a paper that is currently in preparation for publication.

## Conclusions

This study used data from a Japanese health insurance database to demonstrate that, while the prevalence of gout and asymptomatic hyperuricemia was much lower in this pediatric population than among adults, the prevalence clearly increased with age prior to adulthood. Our study also confirmed that in pediatric patients the presence of gout or asymptomatic hyperuricemia may be associated with underlying diseases such as cardiovascular or kidney diseases, congenital disorders, genetic disorders, or metabolic syndrome and its associated diseases. The number of patients with comorbid metabolic syndrome and associated lifestyle diseases increased after puberty. Our findings confirmed the presence of gouty arthritis, gout, and asymptomatic hyperuricemia in pediatric patients, and showed that uric acid lowering drugs are being prescribed to those patients even though the drugs have not yet been approved for pediatric indications. Such off-label use emphasizes the potential need for appropriately tested and approved therapeutic agents in pediatric patients with gout or asymptomatic hyperuricemia.

## Additional file

**Additional file 1 **: **Table S1** List of definitions. **Table S2** Comorbidities coded under the ICD10 coding system (prevalence ≥ 10%) in patients diagnosed with gout and asymptomatic hyperuricemia (n = 276)

## Data Availability

The data set analyzed for this study is not currently available for data sharing.

## Abbreviations

ACE: Angiotensin-converting enzyme
ARB: Angiotensin II receptor blocker
IQR: Interquartile range
SD: Standard deviation
