# Supplementary material for: Prevalence of gout and asymptomatic hyperuricemia in the pediatric population: a cross-sectional study of a Japanese health insurance database
Source: BMC Pediatr. 2020 Oct 15;20:481. doi: 10.1186/s12887-020-02379-0 (PMC7559194; doi:10.1186/s12887-020-02379-0)
Supplement: Supplementary file 1 — Additional file 1 : Table S1 List of definitions. Table S2 Comorbidities coded under the ICD10 coding system (prevalence ≥ 10%) in patients diagnosed with gout and asymptomatic hyperuricemia (n = 276) [file 12887_2020_2379_MOESM1_ESM.pdf]

Additional file 1

Prevalence of gout and asymptomatic hyperuricemia in the pediatric population: a cross-sectional study of a Japanese health insurance database

Contents:

Table S1 List of definitions **2**

Table S2 Comorbidities coded under the ICD10 coding system (prevalence ≥ 10%) in patients diagnosed with gout and asymptomatic hyperuricemia (*n* = 276) **7**

Table S1 List of definitions

Gout

Patients for whom any of the following applied for the period from April 2016 to March 2017.

- Code M10 (Gout) under the ICD10 coding system twice or more in separate months
- Code M10 (Gout) under the ICD10 coding system once or more, and one or more prescriptions for M04 (Antigout preparations) by ATC code and gout diagnosis shown on the same insurance claim form.
- Code M10 (Gout) under the ICD10 coding system once or more, and one or more prescriptions for M01A1 (Anti-rheumatics, non-steroidal plain) or H02A2 (Oral corticosteroids, plain) by ATC code and gout diagnosis shown on the same insurance claim form.

Satisfying the above criteria and with no instances of Code M352 (Behçet's disease) under the ICD10 coding system or Code 8831283 (familial Mediterranean fever) under Standard disease code.

Asymptomatic hyperuricemia

Patients for whom the definition of gout did not apply and any of the following applied for the period from April 2016 to March 2017.

- Code E790 (Hyperuricaemia without signs of inflammatory arthritis and tophaceous disease) under the ICD10 coding system twice or more in separate months.
- Code E790 (Hyperuricaemia without signs of inflammatory arthritis and tophaceous disease) under the ICD10 coding system once or more, and one or more prescriptions for M04 (Antigout preparations) by ATC code and diagnosis for hyperuricaemia without signs of inflammatory arthritis and tophaceous disease shown on the same insurance claim form.

Drug treatment with uric acid lowering drug for gout patients

Patients for whom the following applied for the period from April 2016 to March 2017.

- Satisfying the definition of gout, with code M10 (Gout) under the ICD10 coding system once or more, and one or more prescriptions for M04 (Antigout preparations excluding colchicine) by ATC code and gout diagnosis shown on the same insurance claim form.

Drug treatment with uric acid lowering drug for asymptomatic hyperuricemia patients

Patients for whom the definition of drug treatment with uric acid lowering drugs for gout did not apply and any of the following applied for the period from April 2016 to March 2017.

- Satisfying the definition of asymptomatic hyperuricemia, with code E790 (Hyperuricaemia without signs of inflammatory arthritis and tophaceous disease) under the ICD10 coding system once or more, and one or more prescriptions for M04 (Antigout preparations excluding colchicine) by ATC code and diagnosis for hyperuricaemia without signs of inflammatory arthritis and tophaceous disease shown on the same insurance claim form.

Malignant tumor

Patients for whom the following condition applied for the period from April 2016 to March 2017.

- Code C00-C97 (Malignant neoplasm) or D00-D09 (In situ neoplasms) under the ICD10 coding system

Comorbidities

Coded by ICD10 coding system once or more from April 2016 to March 2017.

- Kidney disease: N00-N08 (Glomerular diseases), N10-N16 (Renal tubulo-interstitial diseases), N17-N19 (Renal failure), N20-N23 (Urolithiasis), N25-N29 (Other disorders of kidney and ureter), I12 (Hypertensive renal disease), I15 (Secondary hypertension), Q60-Q64 (Congenital malformations of the urinary system)
- Cardiovascular disease: I05-I09 (Chronic rheumatic heart diseases), I11 (Hypertensive heart disease), I20-I25 (Ischemic heart diseases), I26-I28

(Pulmonary heart disease and diseases of pulmonary circulation), I30-I52 (Other forms of heart disease), I70-I79 (Diseases of arteries, arterioles and capillaries), Q20-Q28 (Congenital malformations of the circulatory system)
- Metabolic syndrome: E10-E14 (Diabetes mellitus), R73 (Elevated blood glucose level), E66 (Obesity), E78 (Disorders of lipoprotein metabolism and other lipidaemias)
- Down syndrome: Q90 (Down's syndrome)
- Lesch-Nyhan syndrome: E791 (Lesch-Nyhan syndrome)

Treatment

Coded for receipt of treatment once or more from April 2016 to March 2017.

-Dialysis: 114003610 (Additional fee for second or third time guidance <guidance fee for ambulatory peritoneal dialysis>), 114003510 (Guidance fee for ambulatory peritoneal dialysis), 114009310 (Guidance fee for in-home hemodialysis), 114009410 (Additional fee for second or third time guidance <guidance fee for in-home hemodialysis>), 114006610 (Automated peritoneal dialysis system fee), 140029850 (Continuous slow hemofiltration), 140053670 (Additional fee for the disabled etc. <continuous slow hemofiltration>), 140051110 (Artificial kidney, chronic maintenance dialysis [for 5 hours or more]); 140051010 (Artificial kidney, chronic maintenance dialysis [for 4-5 hours]), 140036710 (Artificial kidney, chronic maintenance dialysis [for less than 4 hours]), 140052810 (Artificial kidney, chronic maintenance diafiltration [complicated]), 140007910 (Additional fee for after-hour/holiday <artificial kidney>), 140007710 (Artificial kidney [other]), 140033770 (Additional fee for the disabled etc. <artificial kidney>), 140008170 (Additional fee of induction period <artificial kidney>), 140052570 (Additional fee for ensuring dialysate quality [1] <artificial kidney>), 140052970 (Additional fee for ensuring dialysate quality [2] <artificial kidney>), 140055970 (Additional fee for patient management for lower extremity peripheral arterial disease <artificial kidney>), 140054950 (Additional fee of induction period for 15-30 days, for infant/preschool child <peritoneal dialysis>), 140054850 (Additional fee of induction period [14 days] for infant/preschool child <peritoneal dialysis>), 140008770

(Additional fee of induction period [14 days] <peritoneal dialysis>), 140008670 (Additional fee for abdominal catheter placement <peritoneal dialysis, continuous ambulatory>), 140008510 (Peritoneal dialysis, continuous ambulatory), 140008810 (Peritoneal dialysis [other])

Drugs

- Cardiovascular disease drug: ATC code C01 (Cardiac therapy)
- Diuretic drug: ATC code C03 (Diuretics)
- β blocker: ATC code C07 (beta blocking agents)
- Ca antagonist: ATC code C08 (Calcium antagonists)
- ACE inhibitor and/or ARB: ATC code C09 (Agents acting on the renin-angiotensin system)
- Antihyperlipidemic drug: ATC codes C10 (Lipid regulating/anti-atheroma preparations)
- Antidiabetic drug: ATC code A10 (Drugs used in diabetes)
- Immunosuppressant: ATC code L04X (Other immunosuppressants)
- Vitamin D: ATC code A11C2 (Vitamin D)
- Oral corticosteroid*: ATC code H02A2 (Oral corticosteroids, plain)
- NSAID*: ATC code M01A1 (Anti-rheumatics, non-steroidal plain)
- Colchicine*: colchicine (generic name)
- Uric acid lowering drug†: ATC code M04 (Antigout preparations); note that colchicine is excluded

* Use is defined as follows: Code M10 (Gout) under the ICD10 coding system once or more, and one or more prescriptions for each ATC code and diagnosis shown on the same insurance claim form during the survey period.

† Use is defined as follows: Code M10 (Gout) or E790 (Hyperuricaemia without signs of inflammatory arthritis and tophaceous disease) under the ICD10 coding system once or more, and one or more prescriptions for each ATC code and diagnosis shown on the same insurance claim form is given during the survey period.

Gouty arthritis

Code M10 (Gout) by ICD10 coding system once or more, and diagnosis for gout and prescriptions for ATC codes M01A1 (Anti-rheumatics, non-steroidal plain) or H02A2 (Oral corticosteroids, plain), or colchicine (generic name) shown on the same insurance claim form.

Confirming of intervals between prescriptions for the above-mentioned drugs; if the same drug is prescribed a second time after a no-prescription interval of 14 days or more, that will be counted as a new attack of gouty arthritis.

ICD10 International statistical classification of diseases and related health problems 10th revision, *ATC* anatomical therapeutic chemical classification system, *ACE* angiotensin-converting enzyme, *ARB* angiotensin II receptor blocker, *NSAID* nonsteroidal antiinflammatory drug

Table S2 Comorbidities coded under the ICD10 coding system (prevalence ≥ 10%) in patients diagnosed with gout and asymptomatic hyperuricemia (n = 276)

ICD10 code	Comorbidity [a]	Prevalence (n)
J30	Vasomotor and allergic rhinitis	52.5% (145)
J06	Acute upper respiratory infections of multiple and unspecified sites	42.8% (118)
J20	Acute bronchitis	40.2% (111)
H52	Disorders of refraction and accommodation	38.8% (107)
A09	Diarrhoea and gastroenteritis of presumed infectious origin	35.5% (98)
J45	Asthma	34.4% (95)
L30	Other dermatitis	28.6% (79)
E78	Disorders of lipoprotein metabolism and other lipidaemias	27.5% (76)
H10	Conjunctivitis	27.2% (75)
J02	Acute pharyngitis	27.2% (75)
L85	Other epidermal thickening	24.3% (67)
I10	Essential (primary) hypertension	21.0% (58)
D50	Iron deficiency anaemia	20.7% (57)
K76	Other diseases of liver	20.3% (56)
K59	Other functional intestinal disorders	19.9% (55)
K29	Gastritis and duodenitis	18.5% (51)
J00	Acute nasopharyngitis [common cold]	17.0% (47)
J01	Acute sinusitis	17.0% (47)
L20	Atopic dermatitis	16.7% (46)
J10	Influenza due to other identified influenza virus	15.6% (43)
L70	Acne	14.1% (39)
G40	Epilepsy	13.0% (36)
G47	Sleep disorders	13.0% (36)
I50	Heart failure	12.7% (35)
N28	Other disorders of kidney and ureter, not elsewhere classified	12.7% (35)

E14	Unspecified diabetes mellitus	12.0% (33)
E66	Obesity	11.6% (32)
J32	Chronic sinusitis	11.6% (32)
H60	Otitis externa	11.2% (31)
H61	Other disorders of external ear	11.2% (31)
J11	Influenza, virus not identified	11.2% (31)
E87	Other disorders of fluid, electrolyte and acid-base balance	10.9% (30)
R51	Headache	10.9% (30)
E86	Volume depletion	10.1% (28)

[a] Coded by ICD10 coding system once or more from April 2016 to March 2017.

ICD10 International statistical classification of diseases and related health problems 10th revision
